# High SPARC Expression Starting from Dysplasia, Associated with Breast Carcinoma, Is Predictive for Bone Metastasis without Enhancement of Plasma Levels

**DOI:** 10.3390/ijms161225997

**Published:** 2015-11-26

**Authors:** Paola Maroni, Paola Bendinelli, Daniele Morelli, Lorenzo Drago, Alessandro Luzzati, Giuseppe Perrucchini, Chiara Bonini, Emanuela Matteucci, Maria Alfonsina Desiderio

**Affiliations:** 1 Istituto Ortopedico Galeazzi, Scientific Institute for Research, Hospitalization and Helth Care (IRCCS), 20161 Milano, Italy; paola.maroni@grupposandonato.it (P.M.); lorenzo.drago@unimi.it (L.D.); sandroluzzati@tiscali.it (A.L.); pergiudoc@yahoo.com (G.P.); 2 Dipartimento di Scienze Biomediche per la Salute, Molecular Pathology Laboratory, Università degli Studi di Milano, 20133 Milano, Italy; paola.bendinelli@unimi.it (P.B.); emanuela.matteucci@unimi.it (E.M.); 3 Department of Pathology and Laboratory Medicine, Fondazione IRCCS Istituto Nazionale dei Tumori, 20133 Milano, Italy; daniele.morelli@istitutotumori.mi.it (D.M.); chiara.bonini@istitutotumori.mi.it (C.B.)

**Keywords:** bone metastasis, dysplasia, SPARC, Endothelin 1, ET_A_R, breast carcinoma

## Abstract

In order to become established in the skeleton, metastatic cells disseminating from the breast carcinoma need to acquire organ-specific traits. There are no effective predictors for who will develop bone metastasis to guide long-term predictive therapy. Our purpose was to individuate events critical for bone colonization to make a molecular classification of breast carcinoma useful for bone-metastasis outcome. In dysplasia adjacent to carcinoma and in pair-matched specimens of bone metastasis we examined SPARC expression and localization as well as Endothelin 1/ET_A_R signals by immunohistochemistry, and the evaluation of plasma levels of SPARC by ELISA was also performed. In patients with breast carcinoma metastasizing to bone, SPARC and Endothelin 1/ET_A_R axis were highly expressed from dysplasia until bone metastasis, but the SPARC plasma level was as low as that of normal women, in contrast to patients that never develop bone metastasis, suggesting that circulating SPARC was counter adhesive. Altogether, the early identification of SPARC/Endothelin 1/ET_A_R in dysplastic lesions would be important to devise therapies preventing metastasis engraftment, since often carcinoma cells spread to distant organs at the time or even before patients present with cancer.

## 1. Introduction

Secreted protein acidic and rich in cysteine (SPARC) belongs to the matricellular group of proteins, that are transiently secreted to the Extracellular matrix (ECM) without becoming part of the ECM mesh. Acting at the interface between the cell surface and the ECM, matricellular proteins regulate cellular processes such as adhesion, migration, proliferation and differentiation [1,2,3]. SPARC, also known as basement membrane-40 (BM-40) tumour protein or osteonectin, is a 32–35 kDa multifunctional ECM protein, with affinity for collagens and calcium ions; a high molecular weight form in the bone (43 kDa) is due to glycosylation [4].

The role of SPARC in tumorigenesis is controversial, and some investigations report a positive relationship between elevated levels of SPARC and more aggressive cancers, with poorer clinical outcome, even if this largely depends on the tumour type [5,6]. While normal mammary tissue has undetectable or lightly detectable amounts of SPARC, and benign breast lesions are weakly positive, 75% of both *in situ* and invasive breast carcinomas are strongly positive for SPARC in stromal cells (CD-34-negative, α-SMA-positive) [7,8]. Moreover, SPARC is associated with clinicopathological parameters such as histological differentiation, tumour size, depth of invasion, lymph node metastasis, distant metastasis, and Tumor-Node-Metastasis (TNM) stage [6].

Metastatic occurrence in the skeleton, one of the preferential secondary sites, is the leading cause of death for breast carcinoma, and bone metastases develop also ten years after surgical removal of the primary tumour, since osteotropic malignant cells may have a long period of quiescence before secondary outgrowth [9]. Complications of bone metastases include pain, increased risk of fracture, hypercalcemia and decreased levels of blood cell count. Having reached the bone, malignant cells disrupt the remodelling process that normally occurs. Osteoclast number increases, stimulating bone resorption and the release of factors important for metastasis outgrowth, and osteoblast number might decrease in case of osteolytic bone metastasis while increasing in the osteoblastic or mixed type [3,9].

Improved knowledge of the biochemical processes underlying bone metastasis, has prompted investigation whether skeletal events in patients may correlate with levels of serum or urine markers of bone turnover, thus facilitating early detection and screening for such events. The prevention and the development of therapeutic strategies against bone metastasis implicate the understanding of the carcinoma cell preference to metastasize in the bone tissue. The bone microenvironment may provide growth stimulating factors or other proteins that induce preferential adhesion and proliferation. Based on transgenic models of breast cancer, SPARC expression could be used as a potential prognostic biomarker of tumour severity and/or aggressiveness [10].

As already mentioned, the survival rate in the case of patients with bone metastasis from breast carcinoma is very low. The understanding of molecular mechanisms of organotropism would help the prevention of metastasis, and the establishment of a valid therapy in order to improve the patient’s quality of life and to increase survival. Until now there are only few studies regarding breast carcinoma that tried to disclose the roles of SPARC in bone metastasis.

We have demonstrated a favouring function of endogenous SPARC for bone metastasis from breast cancer using a xenograft model, prepared with clone 1833 derived from MDA-MB231 human breast carcinoma cells. SPARC was induced by Endothelin 1 in 1833 cells with bone tropism, but not in parental cells, and the expression of the two biological stimuli is orchestrated by DNA methyltransferases. Of note, SPARC and Endothelin 1 are expressed in the bone marrow of the bone metastasis xenograft model [11].

The bone marrow is a common homing organ, and reservoir for disseminated tumour cells from breast cancer. Although only a small fraction of patients present overt bone marrow metastasis, 70% of advanced breast cancer patients develop metastasis in the bone [12]. Bone marrow is a source of mesenchymal stem cells that can give rise to cells of mesodermal lineages, such as osteocytes [13]. These mesenchymal stem cells play an important pro-tumorigenic role in the microenvironment of bone metastasis [14], and are also known as bone marrow stromal fibroblasts expressing SPARC/osteonectin [15], that home to sites of tumorigenesis and integrate in primary tumour stroma [13].

Recent research is devoted to understand critical events in breast cancer progression to make a molecular classification. Until now the breast carcinomas are classified as Basal and Luminal, and the latter in A and B principally on Ki67 value [16]. Ki67 is a molecular marker expressed in all cell-cycle phases except G0; the optimal Ki67 level discriminating Luminal A from B is 13.2% [17].

Our hypothesis was that SPARC might be a key molecular signal released from primary breast carcinoma and/or locally produced in bone metastasis to influence the bone niche formation in the secondary site. It has been proposed that bone colonization is initiated in a microenvironment niche exhibiting active osteogenesis, and heterotypic adherens junctions take place between metastatic and osteogenic cells [18]. In this context, the present paper deals with the evaluation of SPARC expression during ductal breast carcinoma progression, from the dysplastic lesion until bone metastasis establishment: SPARC distribution in neoplastic cells *versus* stroma, and the plasma levels were examined. Concomitantly, the expression of Endothelin 1 and of its receptor ET_A_R were assayed to clarify whether the Endothelin 1/ET_A_R pattern completed SPARC findings as predictive value for bone metastasis. Activation of autocrine and paracrine signalling by Endothelin 1 binding to its receptors elicits pleiotropic effects on tumour cells and on the host microenvironment for immunomodulation and intravasation [19]. Also, Endothelin 1 and ET_A_R are expressed in breast carcinoma metastasizing to bone [20], even if the predictive value of this axis has not been clarified until now.

The aim of the present paper was to individuate molecular events to extend the classification of invasive ductal carcinoma, adding predictive markers for the outcome of breast cancer patients related to the development of bone metastasis.

The results indicate that the biological function of SPARC depended on the phase of breast cancer progression, the tissue compartment in which it was expressed, and its body distribution, with plasma release of SPARC being opposite to metastasis engraftment. SPARC was highly expressed in the stroma of bone metastasis, suggesting a positive effect on the outgrowth also due to a cross talk with metastatic cells expressing SPARC. The association of SPARC with Endothelin 1 and ET_A_R—starting from dysplasia until the end of the metastatic process—appears to be of fundamental importance as a predictive index of bone metastasis formation.

## 2. Results

### 2.1. SPARC Expression, Distribution and Intracellular Localization during Breast Carcinoma Progression

To clarify whether SPARC had a differential involvement in the various phases of breast carcinoma progression, we evaluated SPARC expression in dysplastic lesions, primary ductal carcinoma and bone metastasis using human specimens pair-matched (*n* = 5). We took into consideration the distribution in tissue compartments, the intracellular localization and the cell types expressing SPARC.

**Figure 1:**
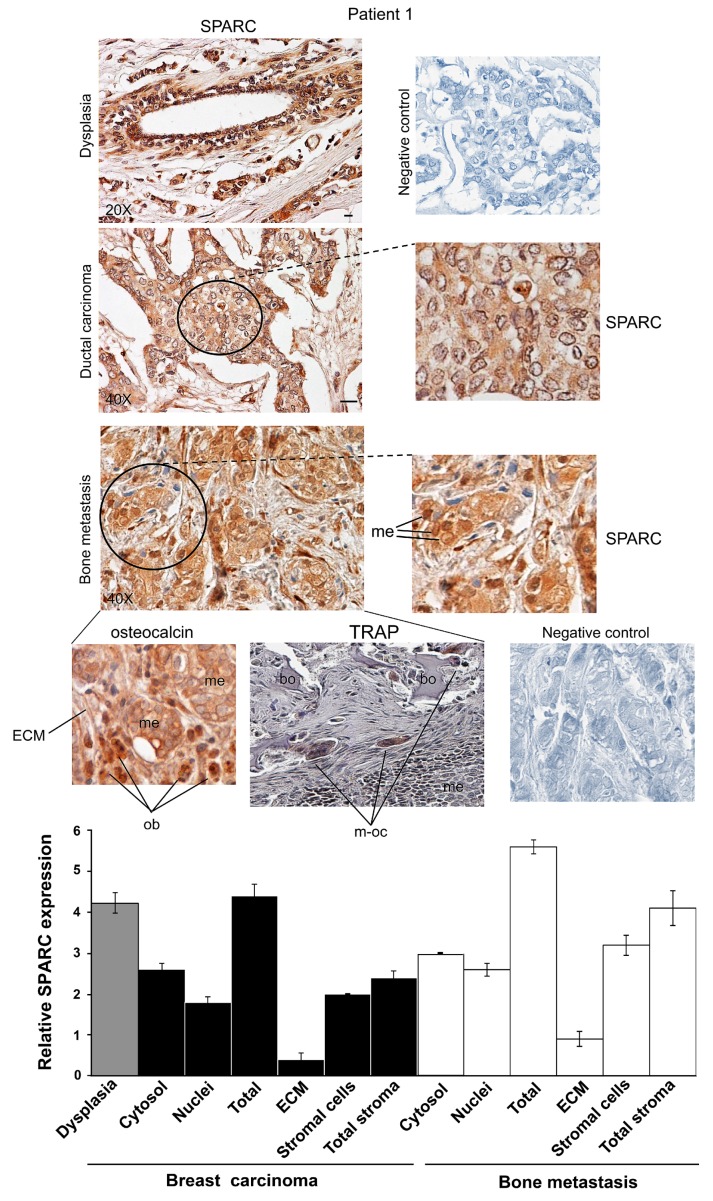
SPARC expression during ductal breast carcinoma progression from dysplasia until bone metastasis for Patient 1. Representative images of immunohistochemical staining for SPARC and osteocalcin were shown. In bone metastasis, TRAP assay was also performed. Magnifications of details are shown. Bar = 100 μm. ECM, Extracellular matrix; me, metastatic cells; bo, bone; ob, osteoblasts; m-oc, mature osteoclasts. Negative controls were assayed without the specific antibody. The histograms, showing the relative values for SPARC expression, were prepared by using the score values reported in Supplementary Table S1. The data are the means ± S.E. of experiments performed in triplicate.

**Figure 2:**
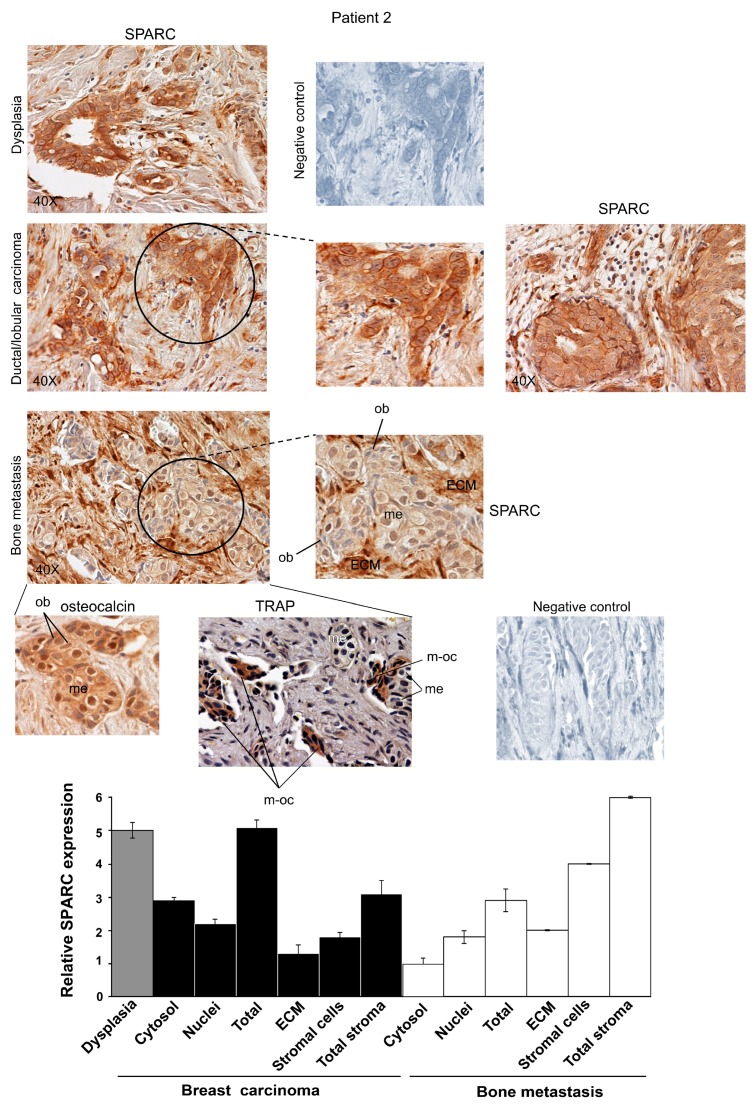
SPARC expression during ductal/lobular breast carcinoma progression from dysplasia until bone metastasis for Patient 2. Representative images of immunohistochemical staining for SPARC and osteocalcin were shown. In bone metastasis, TRAP assay was also performed. Magnifications of details are shown. ECM, Extracellular matrix; me, metastatic cells; ob, osteoblasts; m-oc, mature osteoclasts. Negative controls were assayed without the specific antibody. The histograms, showing the relative values for SPARC expression, were prepared by using the score values reported in Supplementary Table S1. The data are the means ± S.E. of experiments performed in triplicate.

**Figure 3:**
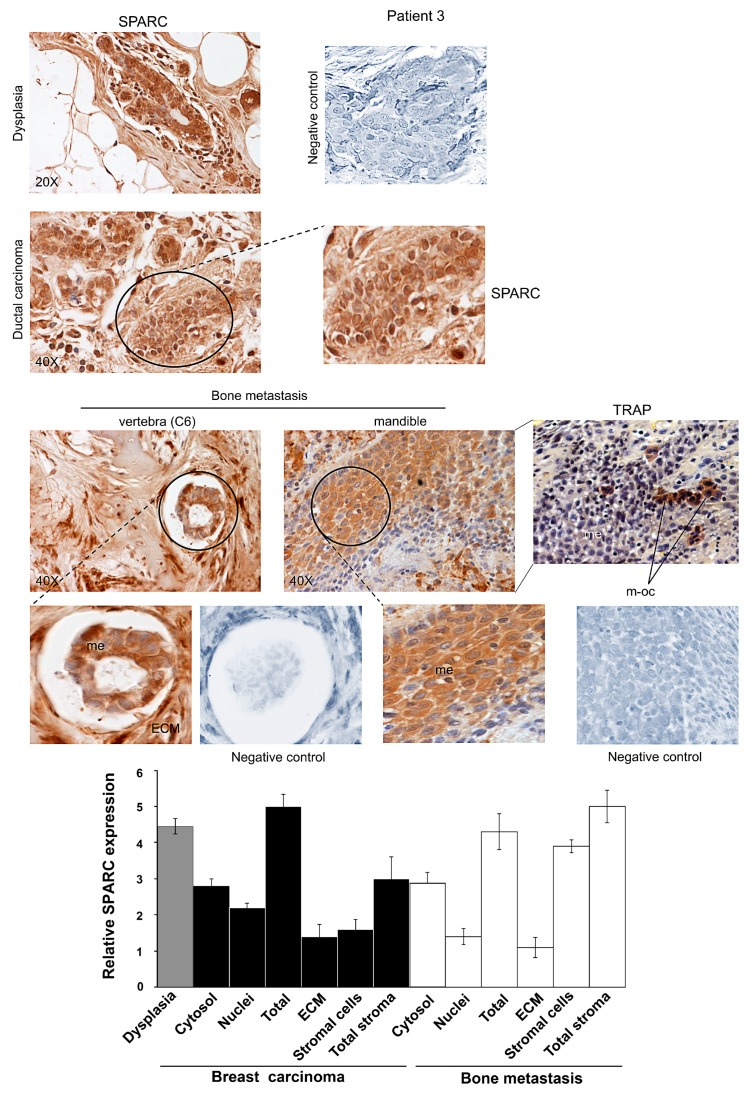
SPARC expression during ductal breast carcinoma progression from dysplasia until bone metastasis for Patient 3. Representative images of immunohistochemical staining for SPARC were shown. In bone metastasis established in the mandible, TRAP assay was also performed. Magnifications of details are shown. ECM, Extracellular matrix; me, metastatic cells; m-oc, mature osteoclasts. Negative controls were assayed without the specific antibody. The histograms, showing the relative values for SPARC expression, were prepared by using the score values reported in Supplementary Table S1. The data are the means ± S.E. of experiments performed in triplicate.

Representative images for Patients 1, 2 and 3 are shown in Figure 1, Figure 2 and Figure 3. The Patients 1 and 3 had invasive ductal carcinoma, and Patient 2 had an invasive ductal/lobular (mixed) carcinoma: all the three Patients developed metastasis within 7–16 years. As shown in Supplementary Table S1, the percentage of positive cells for SPARC signal was estimated by analyzing 100 cells per field in 10 fields, and the immunoreactivity score was indicated [21]. We examined the localization and degree of SPARC expression in cytosol and nuclei of carcinoma and metastatic cells, and in primary tumour and metastasis stroma compartments, *i.e.*, ECM and supportive cells, and the semiquantitative evaluation was made and used for the histograms of Figure 1, Figure 2 and Figure 3.

All the three Patients showed extraordinary similarities in SPARC expression: a strong immunostaining occurred in epithelial-dysplastic cells and in breast carcinoma cells. In the latter, SPARC almost equally localized in the cytosol and the nuclei. As regards primary tumour stroma, the ECM had lower levels of the matricellular protein than the supportive cells; this was particularly evident in Patient 1. Thus, in primary carcinoma SPARC distributed principally in tumour cells, compared to the stroma. For Patient 2 (Figure 2), the expression of SPARC occurred both in infiltrated-invasive carcinoma (left panel) and in the infiltrating-lobular component (right panel).

In bone metastases of Patients 1, 2 and 3, SPARC was highly expressed in stromal cells, while being variously expressed in metastatic cells within the cytosol. For Patient 3, we showed SPARC immunostaining for bone metastases established in the vertebra (C6) and in the mandible.

In addition, cells morphologically different from metastatic cells were also positive for SPARC in all the samples of bone metastasis tissues for Patients 1, 2 and 3; SPARC negative cells (blue) were evident after SPARC immunostaining (Figure 1, Figure 2 and Figure 3). We tried to identify these SPARC negative cells by immunohistochemistry analysis of osteocalcin, an osteoblast marker [22], and by tartrate-resistant acid phosphatase (TRAP), index of osteoclasts [23]. As shown in Figure 1 and Figure 2, the bone metastasis contained osteocalcin-positive cells, *i.e.*, osteoblasts (index of osteogenesis). In addition, osteocalcin was expressed by metastatic cells (index of osteomimicry). Concomitantly, in serial sections we showed mature osteoclasts (m-oc) on the basis of the number of nuclei (more than three) and TRAP activity: m-oc were shown in the areas of osteolysis, with presence of bone (bo) residues (Figure 1 and Supplementary Figure S1). In Figure 3, the invasive leading edge of bone metastasis also contained TRAP-positive mature osteoclasts, which seemed to correspond in part to the cells that remained blue (unstained) after SPARC immunostaining.

**Figure 4:**
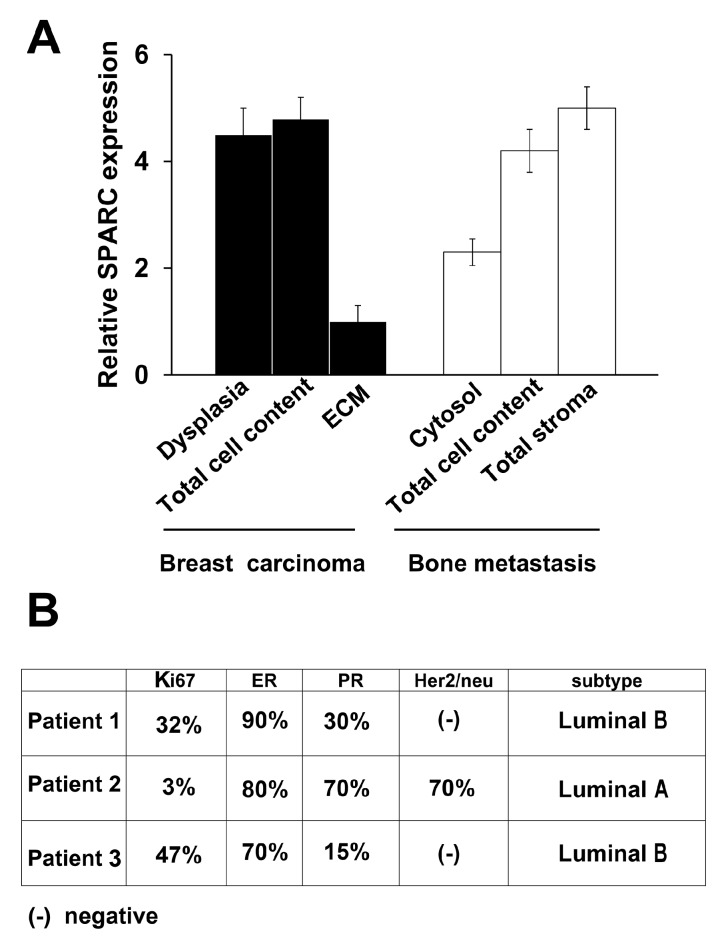
SPARC values and Classification of Patients 1, 2 and 3. (**A**) For Patients 1–3, the mean values for SPARC (*n* = 3) were shown, and the statistical evaluation with the analysis of variance (one way ANOVA) has been performed. F = 9.92; *p* < 0.001; and (**B**) The Classification for Patients 1, 2 and 3 has been reported. The Patient 2 had a ductal/lobular carcinoma: The ductal and the lobular components were Her2/neu positive and negative, respectively.

The statistical evaluation of the mean values for the Patients 1–3 has been reported in Figure 4A. The parameters under investigation for SPARC expression showed remarkable differences, indicating that SPARC might have different functions in the various phases of breast carcinoma progression with metastasis outcome.

The three Patients positively correlated for SPARC signal in dysplasia, total cell content and stroma of metastasis. The SPARC signal in the ECM of carcinoma tissue had the lowest value in respect to that of all the other parameters.

Figure 4B shows breast carcinoma subtypes for Patients 1, 2 and 3, based on Ades *et al.* [17]. Patients 1 and 3 were classified as Luminal B, without Her2/neu, with lower expression of progesterone receptor (PR) and a higher value of Ki67 (proliferation gene) in respect to Patient 2 (ductal component). Patient 2 was characterized as having the highest combined expression of oestrogen receptor (ER) plus PR, with positive expression of Her2/neu in the ductal component of the breast carcinoma, that was classified as Luminal A.

Altogether, this classical molecular classification did not seem suitable enough to predict the formation of bone metastasis. The analysed patients without bone metastases, belonged to Luminal A more than B subtype (Supplementary Table S2).

Therefore, further studies were undertaken to make a molecular classification that considered molecular events critical for invasive breast carcinoma to develop bone metastasis.

### 2.2. SPARC as Predictive Marker of Bone Metastasis: Association with Plasma Levels of SPARC and Tissue Endothelin 1/ET_A_R

**Figure 5:**
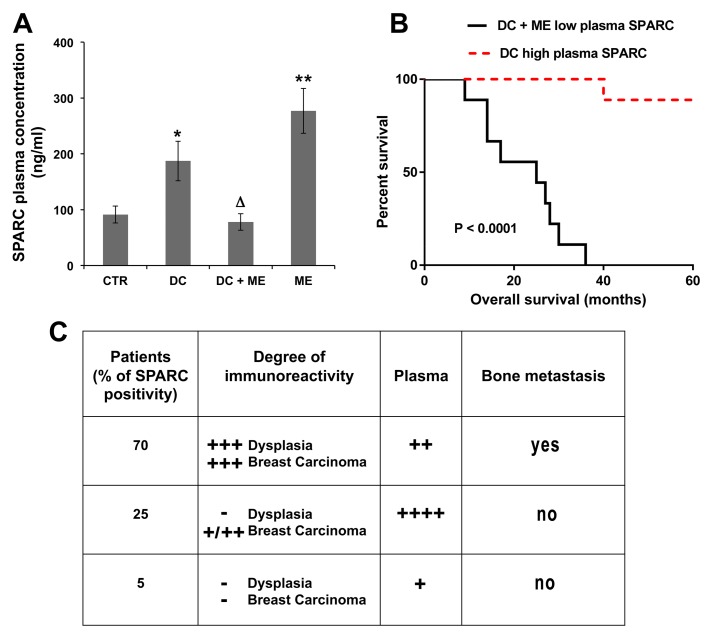
Plasma levels of SPARC and predictivity of bone metastasis. (**A**) ELISA assay was performed using the plasma samples from control (CTR), ductal carcinoma-bearing patients (DC), with or without bone metastasis (ME). The data are the means ± S.E. of nine samples, assayed in triplicate; * *p* < 0.05 and ** *p* < 0.005 versus CTR; Δ *p* < 0.05 versus DC; (**B**) Survival curve on Kaplan-Meier plots of the data from breast carcinoma patients with or without bone metastasis development; and (**C**) The Table shows the percentage of positivity for SPARC at the degree indicated (second column); the plasma value score was also shown (third column), and the relationship with bone metastasis formation is indicated (fourth column). − (negative signal); + (weak signal); ++ (moderate signal); +++ (strong signal); ++++ (very strong signal).

**Figure 6:**
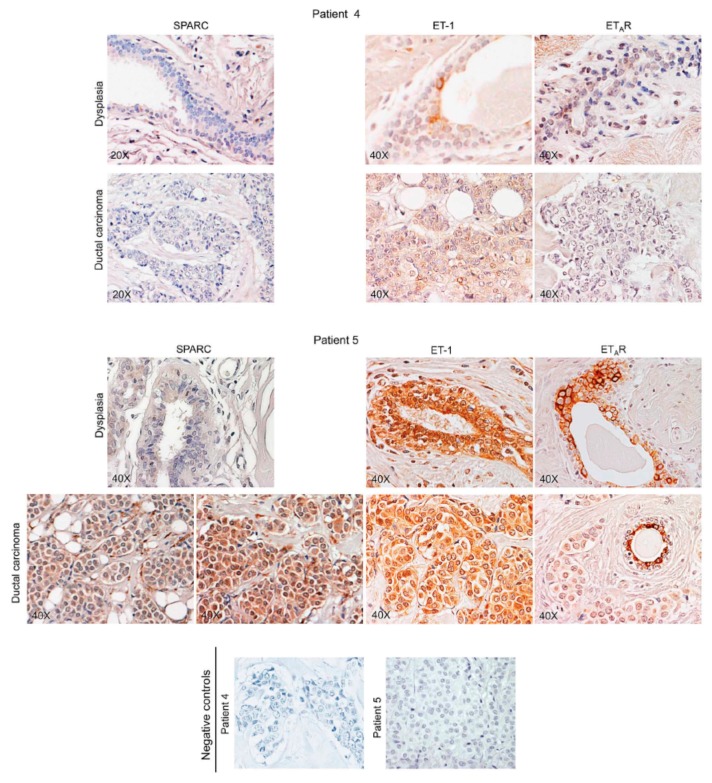
SPARC and Endothelin 1 (ET-1)/ET_A_R in tissue specimens from patients without bone metastasis development. For Patients 4 and 5, representative images of immunohistochemical staining for SPARC and ET-1/ET_A_R in dysplastic lesions, associated with ductal breast carcinoma, are shown. Negative controls were assayed without the specific antibody.

**Figure 7:**
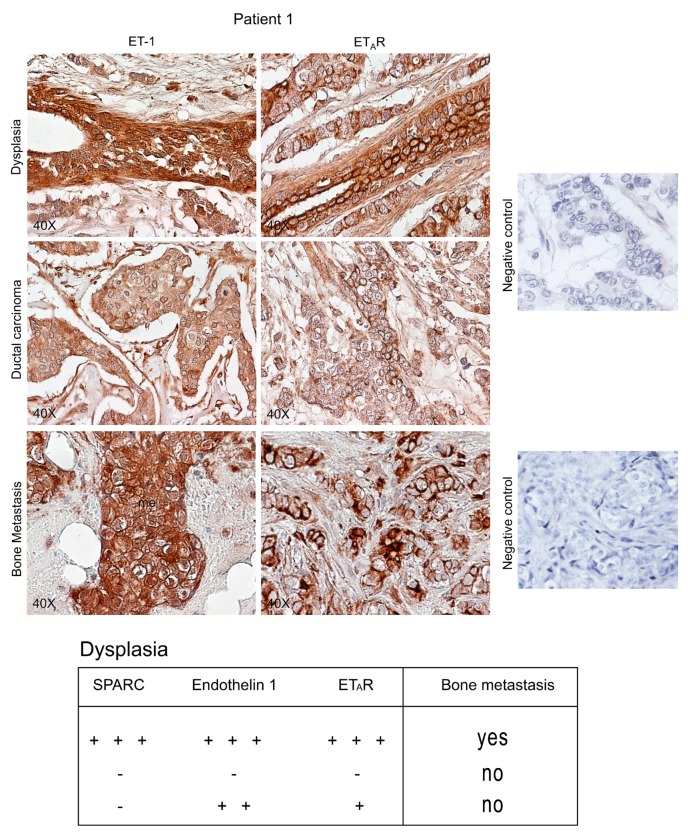
Endothelin 1 (ET-1)/ET_A_R in tissue specimens from Patient 1 during breast carcinoma progression until bone metastasis. Representative images of immunohistochemical staining for ET-1 and ET_A_R in dysplastic lesions, associated with ductal breast carcinoma, and in bone metastasis are shown. Negative controls were assayed without the specific antibody. The Table reports the indicative predictability of SPARC combined with ET-1/ET_A_R for metastasis formation in the skeleton. − (negative signal); + (weak signal); ++ (moderate signal); +++ (strong signal).

We evaluated plasma levels of SPARC (Figure 5A), and tissue Endothelin 1/ET_A_R (Figure 6 and Figure 7), to determine whether there was a correlation with bone metastasis formation.

As shown in Figure 5A, SPARC plasma levels in ductal carcinoma (DC) and in bone metastasis (ME)-bearing patients doubled and tripled, respectively, compared to that of normal women (CTR). Notably, in the breast carcinoma-bearing patients who developed metastasis (DC + ME), the plasma level was similar to that of CTR; the blood was collected at the moment of removal of the primary carcinoma from patients not receiving therapy. In Figure 5B, we show the Overall survival curve referred to low and high SPARC expression in the blood of patients with primary breast carcinoma, developing or not bone metastasis, respectively. According to the available information at the time, the patients without bone metastasis survived free of diseases for 60 months, except for one patient who died at 40 months after the surgery, due to ovary/Fallopian tubes carcinoma disseminated to the peritoneum. Bone metastasis bearing-patients, however, died within 36 months after surgical removal of the primary carcinoma.

In Figure 5C, we considered the data obtained until now. A high degree of immunoreactivity for SPARC, as shown in the Table, was present in 70% of breast carcinoma patients examined (3/5), and pair-matched for developing bone metastasis; the plasma level was not elevated.

Certainly, the highest plasma levels of SPARC were those of the patients who did not develop metastasis, and who showed variable positivity for SPARC (from + to ++, *n* = 3) in the breast carcinoma tissue (see representative images of fields for Patient 5, Figure 6). The lowest plasma levels of SPARC were those of SPARC negative primary tumours (*n* = 3), that showed 5% of cases with faint SPARC staining (representative images for Patient 4, Figure 6).

Figure 6 and Figure 7 also report Endothelin 1 and ET_A_R expression. For dysplasia and breast carcinoma of Patients 5 and 4, Endothelin 1/ET_A_R axis showed intermediate (+/++) and negative values, respectively, while Patient 1 showed strong signals (+++/++++) in specimens of dysplastic tissue and bone metastasis, with breast carcinoma staining positively (++). In particular, ET_A_R distribution at plasma membrane level characterizes the dysplastic-epithelial and bone metastatic cells [20].

In conclusion, without associated strong SPARC expression, the Endothelin 1/ET_A_R positivity in dysplastic lesions did not appear to be sufficient as predictive index of bone metastasis (Figure 7, Table). All the five pair-matched patients had SPARC/Endothelin 1/ET_A_R (+++) in dysplastic lesions; as shown in Patients 1–3, the strong signal of SPARC remained persistently elevated in breast carcinoma cells as well as in bone metastasis cells and stroma, while the other two pair-matched patients had a lower staining of SPARC (+) in the carcinoma cells (data not shown).

## 3. Discussion

Here we show that elucidating the molecular basis of ductal breast carcinoma progression might lead to effective ways to predict the bone colonization of metastasis, and to make a valid molecular classification important to devise a preventive therapy.

The ectopic secretion of proteins, known to be microenvironmental stimuli like SPARC and Endothelin 1, appeared to confer osteogenic traits to disseminated breast carcinoma cells, important for the attachment to bone matrix [7,22]. We demonstrated for the first time in bone metastasis of humans that at the site of colonization, SPARC was expressed by metastatic cells as well as by osteoblasts, which were identified on the basis of osteocalcin immunostaining. We suggest that osteoblasts were involved not only in osteogenesis, but also in the recruitment of osteoclasts [22], maintaining an elevated bone turnover with a consequent release of Endothelin 1 in the microenvironment. Thus, the induction of SPARC by Endothelin 1 was likely to occur also in metastatic cells of patients, as reported before for 1833 clone [11]. In addition, SPARC is a marker of differentiation of osteogenic bone cells [22]. Altogether, the events reported here might be critical for microscopic bone metastasis to progress into clinically significant lesions.

Our data are consistent with those obtained by Wang *et al.* with an experimental model [18], in which he demonstrates that bone colonization is initiated by a microenvironment niche exhibiting active osteogenesis. We hypothesize that bone niche evolution requires SPARC. Since human bone metastasis from ductal breast carcinoma shows E-cadherin expression [24], an heterotypic interaction with osteogenic cells would occur, as reported in a human/mouse experimental system, giving a proliferative advantage [18]. We cannot exclude that the observed high SPARC signal in supportive cells of bone metastasis conferred a proliferative advantage to metastatic cells [25], and that the latter producing SPARC competed with stem cell fibroblasts for the niche becoming hospitable [26].

The strength of our study in patients is the analysis of pair-matched invasive ductal breast carcinoma from the early preneoplastic stages until bone metastasis formation. The function of SPARC seems to depend on the microenvironment, in terms of local composition of matrix molecules, cytokines and protease profile, and on whether it is expressed by the malignant cells themselves or by neighboring stromal cells [3].

Our principal finding useful for a molecular classification was the very high expression of SPARC (70% incidence, as bone metastasis) in dysplastic epithelium, associated with ductal breast carcinoma, and in bone metastatic cells and stroma (ECM and supportive cells). Since SPARC is able to organize collagen and other ECM proteins, we suggest that in patients it would be involved in creating a fibrotic environment favorable for colonization, similar to lysyl oxidase (LOX) [27,28]. However, for bone metastasis predictive value, the association of SPARC signal with those of Endothelin 1/ET_A_R seemed essential, starting from their presence in the dysplastic lesions. It is known that cancer cells may disseminate very early, even before patients present with cancer, and might form micrometastasis in a permissive environment of the secondary site such as that of bone. Our hypothesis is that a local production of SPARC in the hospitable bone, possibly regulated by Endothelin 1/ET_A_R axis, would be important for bone niche formation and evolution. Notably, the plasma level of SPARC was not elevated in breast cancer patients with bone metastasis outcome.

Moreover, stem cell fibroblasts seem to migrate to primary carcinoma [13], and we suggest that they were responsible for the release of SPARC to tumor cells. In human specimens, breast carcinoma cells and stroma presented SPARC signals, in agreement with the literature [29]. A recycling of SPARC would occur in the primary breast carcinoma, and a role of exosomes cannot be excluded since these microvescicles can modulate nearby or distant target cells by direct contact of their surface molecules, to activate intracellular pathways. Alternatively, on internalization by membrane fusion or endocytosis, exosomes deliver their protein content [30]. Studies are in progress to evaluate this mechanism, since the study with MDA-MB231 cells indicates that *in vitro* breast carcinoma scarcely produces the matricellular protein SPARC [11]. Consistently, primary breast carcinoma that did not give bone metastasis abundantly release SPARC in the plasma, likely preventing, therefore, the engraftment of disseminated cells in the secondary bone site. In primary tumor, SPARC was likely to exert counter-adhesive roles important for the intermediate state of adhesiveness, and responsible for motility and invasiveness. Except for one, all of these breast carcinoma bearing patients are still alive 60 months after surgery. Circulating tumor cells shed from breast carcinoma, are a heterogeneous population including EpCAM-positive cells and cells with mesenchymal phenotype (EMT), which are studied for the analysis of the therapeutic response to different chemotherapeutic regimens [31].

In the future personalized medicine, the molecular classification based on SPARC associated with Endothelin 1/ET_A_R would be of great importance to devise therapies against bone metastasis with nanoparticles, that target drugs with a SPARC-dependent endosomal mechanism [32].

## 4. Experimental Section

### 4.1. Patient Recruitment

We studied by immunohistochemistry 11 patients bearing invasive ductal breast carcinoma (Patient 2 had a ductal/lobular form): 5 were pair-matched with bone metastasis; 6 did not have bone metastasis. ELISA assay in the plasma of the following groups of women was performed (each with *n* = 9): normal women (control, CTR), ductal carcinoma without bone metastasis (DC), ductal carcinoma with bone metastasis (DC + ME), bone metastasis (ME). DC were examined for clinical classification (Supplementary Table S2).

### 4.2. Immunohistochemistry Assay

We analysed 5 serial sections prepared from human specimens of primary-invasive ductal or mixed breast carcinoma and associated dysplastic lesions, and from pair matched bone metastasis (collected during surgical interventions at Istituto Ortopedico Galeazzi, Milano, Italy). All patients provided informed consent, in accordance with the Declaration of Helsinki. The tissue sections were fixed and decalcified before immunostaining [24]. The following antibodies were used: anti-SPARC (1:50, H-90 from Santa Cruz Biotechnology, Santa Cruz, CA, USA), anti-Endothelin 1 (1:50, H-38 from Santa Cruz Biotechnology), anti-ET_A_R (4 μg/mL, from Abcam, Cambridge, UK), anti-osteocalcin (5 μg/mL, from Abcam). Negative controls were assayed without the specific antibodies. We made a semiquantitative evaluation of the sample after immunostaining, and the scores were: no staining = 0, very weak staining = 1, weak staining = 2, moderate staining = 3, strong staining = 4, very strong staining = 5.

### 4.3. Tartrate-Resistant Acid Phosphatase (TRAP) Staining

The detection of osteoclasts was performed on TRAP-stained paraffin-embedded sections with the use of a commercial kit (Sigma-Aldrich, St. Louis, MO, USA), following manufacturer’s instructions. After incubation for 1 h at 37 °C, the sections were counterstained with hematoxylin, and the osteoclasts were monitored under a microscope (Eclipse 80i; Nikon, Milano, Italy).

### 4.4. ELISA Assay of Plasma SPARC

The plasma levels of SPARC were evaluated on samples prepared at the moment of the removal of the breast carcinoma (Istituto Nazionale dei Tumori, Milano, Italy), and on plasma samples from bone metastasis bearing-patients before surgical removal of the metastatic tissue (Istituto Ortopedico Galeazzi, that also furnished plasma samples of control healthy women). We used the commercial kit Quantikine, human SPARC (R&D System, Abingdon, UK). The O.D. values were measured with Glomax Discover System (Promega, Madison, WI, USA), using the standard curve of the kit.

### 4.5. Statistical Analysis

The semiquantitative data of SPARC expression in tissue samples of breast carcinoma and associated dysplasia, and of bone metastasis were evaluated by the analysis of variance of the means, with *p* < 0.05 considered significant. The survival data were analyzed by Kaplan-Meier method, and the log-Rank (Mantel-Cox) test. *p* < 0.05 was considered significant.

## Supplementary Materials

Supplementary materials can be found at http://www.mdpi.com/1422-0067/16/12/25997/s1.
