# Supplementary material for: High SPARC Expression Starting from Dysplasia, Associated with Breast Carcinoma, Is Predictive for Bone Metastasis without Enhancement of Plasma Levels
Source: Int J Mol Sci. 2015 Nov 26;16(12):28108–22. doi: 10.3390/ijms161225997 (PMC4691022; doi:10.3390/ijms161225997)
Supplement: Supplementary file 1 [file ijms-16-25997-s001.pdf]

Supplementary Information: High SPARC Expression Starting from Dysplasia, Associated with Breast Carcinoma, Is Predictive for Bone Metastasis without Enhancement of Plasma Levels

Paola Maroni, Paola Bendinelli, Daniele Morelli, Lorenzo Drago, Alessandro Luzzati, Giuseppe Perrucchini, Chiara Bonini, Emanuela Matteucci and Maria Alfonsina Desiderio

Table S1. Analysis of the degree of immunoreactivity for SPARC during breast carcinoma progression. Ten fields were analyzed for each specimen, considering the degree of positivity for SPARC signal in 100 cells, and intracellular and tissue localization were evaluated. The semiquantitative score values have been reported.

Patient 1

Specimens	Degree of Immunoreactivity for SPARC											
	**Field 1**	**Field 2**	**Field 3**	**Field 4**	**Field 5**	**Field 6**	**Field 7**	**Field 8**	**Field 9**	**Field 10**	**Mean**	**Tot. Cell Cont.**
Dysplasia (+++)											4.2	
**Breast Carcinoma**	65%	60%	80%	80%	90%	70%	60%	60%	60%	80%		
Cytosol	++	++	++	++	+	+	+	+	++	++	2.6	
Nuclei	+	+/−	+	+	+	+	+/−	+	+	+	1.8	4.4
Stroma (ECM)	+/−	−	+/−	+/−	+/−	−	−	−	−	−	0.4	
Stromal cells	+	+	+	+	+	+	+	+	+	+	2	2.4
**Bone Metastasis**	90%	70%	70%	90%	80%	90%	80%	85%	70%	95%		
Cytosol	++	++	++	++	++	++	++	++	++	++	3	
Nuclei	++	++	++	++	+	+	+	++	++	+	2.6	5.6
Stroma (ECM)	+/−	−	−	+/−	+/−	+/−	+/−	+/−	+	+/−	0.9	
Stromal cells	+++	+++	+++	+++	++	+	+	++	++	++	3.2	4.1

Patient 2

Specimens	Degree of Immunoreactivity for SPARC											
	Field 1	Field 2	Field 3	Field 4	Field 5	Field 6	Field 7	Field 8	Field 9	Field 10	Mean	Tot. Cell cont.
Dysplasia (++++)											5	
**Breast Carcinoma**	80%	70%	65%	80%	70%	70%	60%	60%	65%	75%		
Cytosol	++	++	++	++	++	++	++	++	+	++	2.9	
Nuclei	+	+	+	+	+	+	+	++	++	+	2.2	5.1
Stroma (ECM)	+/−	−	+/−	+	+/−	+	+	+	−	+	1.3	
Stromal cells	+	+/−	+	+/−	+	+	+	+	+	+	1.8	3.1
**Bone Metastasis**	90%	60%	80%	75%	75%	65%	75%	70%	75%	80%		
Cytosol	+/−	+/−	−	+/−	+/−	+/−	+/−	+/−	+/−	+	1	
Nuclei	+	+	+/-	+	+	+	+	+/−	+/−	++	1.8	2.8
Stroma (ECM)	+	+	+	+	+	+	+	+	+	+	2	
Stromal cells	+++	+++	+++	+++	+++	+++	+++	+++	+++	+++	4	6.0

Patient 3

Specimens	Degree of Immunoreactivity for SPARC											
	Field 1	Field 2	Field 3	Field 4	Field 5	Field 6	Field 7	Field 8	Field 9	Field 10	Mean	Tot. Cell cont.
Dysplasia (+++)											5	
**Breast Carcinoma**	60%	75%	75%	85%	60%	65%	70%	75%	60%	60%		
Cytosol	++	++	+++	++	+	++	++	++	+	+	2.8	
Nuclei	+	+	+	++	+	+	++	+	+	+	2.2	5.0
Stroma (ECM)	+	+/−	−	+	+	++	+	+	−	−	1.4	
Stromal cells	+	+	+	+	+	+	−	+	+	−	1.6	3.0
**Bone Metastasis**	80%	95%	70%	80%	85%	75%	85%	70%	75%	90%		
Cytosol	+	++	+	+	+	+++	+++	++	+++	++	2.9	
Nuclei	+/−	+/−	+	+	+	+/−	−	+/−	+	+	1.4	3.0
Stroma (ECM)	+	−	−	−	+	+/−	+	+/−	+/−	+	1.1	
Stromal cells	+++	++++	++	+++	+++	+++	+++	++	+++	+++	3.9	5.0

% = Percentage of positivity; The staining intensity was categorized into five grades from (−) to (++++): − (no staining) = 0; +/− (very weak staining)= 1; + (weak staining) = 2; ++ (moderate staining) = 3; +++ (strong staining)= 4; ++++ (very strong staining) = 5.

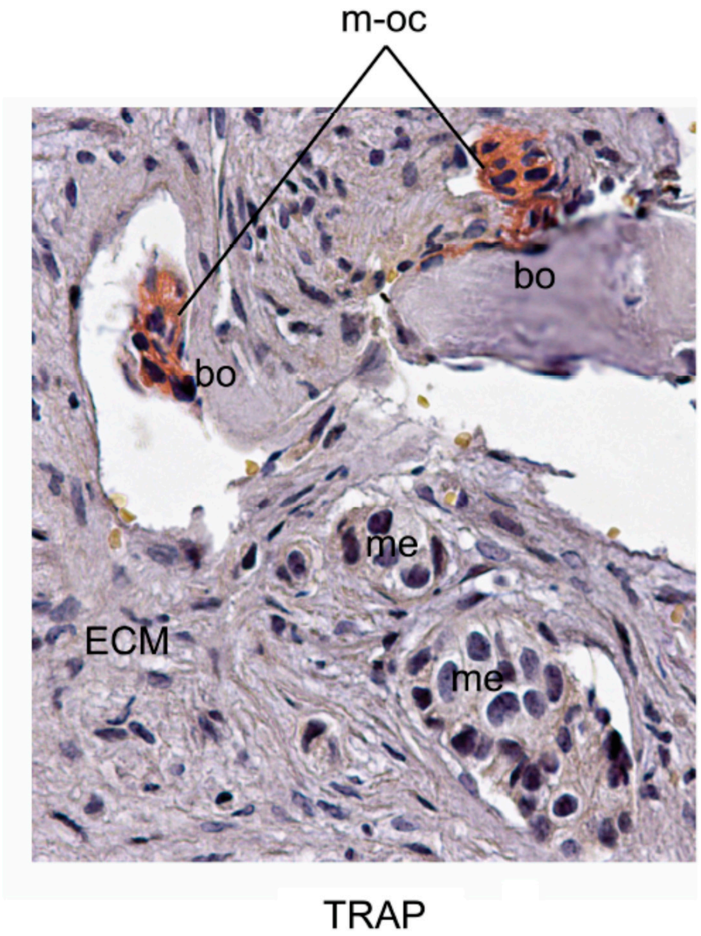

Figure S1. TRAP assay in bone metastasis tissue of Patient 2. Multinucleate mature osteoclasts (m-oc) stained positively for TRAP activity, and were present in areas of osteolysis. bo, bone fragments; me, metastatic cells; ECM, Extracellular matrix.

Table S2. Classification of the patients ($n = 9$) with breast carcinoma without bone metastasis. Ki67 values, and ER and PR scores for Luminal A and B have been shown; Her2/neu was never expressed.

	**Ki67**	**ER**	**PR**	**Her2/neu**	**subtype**
Patient 1	25%	50%	-	-	Luminal B
Patient 2	10%	66%–100%	66%–100%	-	Luminal A
Patient 3	45%	>66%	>66%	-	Luminal B
Patient 4	14%	66%–100%	66%–100%	-	Luminal A
Patient 5	70%	66%	10%	-	Luminal B
Patient 6	45%	50%	15%	-	Luminal B
Patient 7	22%	90%	10%	-	Luminal B
Patient 8	14%	66%–100%	33%–66%	-	Luminal A
Patient 9	20%	66%–100%	66%–100%	-	Luminal B
